# Skin microbiota analysis in patients with anorexia nervosa and healthy-weight controls reveals microbial indicators of healthy weight and associations with the antimicrobial peptide psoriasin

**DOI:** 10.1038/s41598-022-19676-6

**Published:** 2022-09-15

**Authors:** Britt M. Hermes, Franziska Rademacher, Cecilia Chung, Gisa Tiegs, Marie-Christin Bendix, Martina de Zwaan, Jürgen Harder, John F. Baines

**Affiliations:** 1 Max Planck Institute for Evolutionary Biology, Plön, Germany; 2 Section of Evolutionary Medicine, Institute for Experimental Medicine, Kiel University, Kiel, Germany; 3 Lübeck Institute of Experimental Dermatology, University of Lübeck, Lübeck, Germany; 4 Department of Dermatology, University Hospital Schleswig-Holstein, Kiel, Germany; 5 Institute of Experimental Immunology and Hepatology, University Medical Center Hamburg-Eppendorf, Hamburg, Germany; 6 Department of Psychosomatic Medicine and Psychotherapy, Hannover Medical School, Hannover, Germany

**Keywords:** Malnutrition, Microbiome

## Abstract

Anorexia nervosa (AN), a psychiatric condition defined by low body weight for age and height, is associated with numerous dermatological conditions. Yet, clinical observations report that patients with AN do not suffer from infectious skin diseases like those associated with primary malnutrition. Cell-mediated immunity appears to be amplified in AN; however, this proinflammatory state does not sufficiently explain the lower incidence of infections. Antimicrobial peptides (AMPs) are important components of the innate immune system protecting from pathogens and shaping the microbiota. In *Drosophila melanogaster* starvation precedes increased AMP gene expression. Here, we analyzed skin microbiota in patients with AN and age-matched, healthy-weight controls and investigated the influence of weight gain on microbial community structure. We then correlated features of the skin microbial community with psoriasin and RNase 7, two highly abundant AMPs in human skin, to clarify whether an association between AMPs and skin microbiota exists and whether such a relationship might contribute to the resistance to cutaneous infections observed in AN. We find significant statistical correlations between Shannon diversity and the highly abundant skin AMP psoriasin and bacterial load, respectively. Moreover, we reveal psoriasin significantly associates with *Abiotrophia*, an indicator for the healthy-weight control group. Additionally, we observe a significant correlation between an individual’s body mass index and *Lactobacillus*, a microbial indicator of health. Future investigation may help clarify physiological mechanisms that link nutritional intake with skin physiology.

## Introduction

Anorexia nervosa (AN) is a psychiatric condition typically affecting females with an estimated lifetime prevalence between 0.5 and 2.0% and a peak age in onset between 13 and 18 years of age^1^. The hallmark feature of AN is low body weight for age and height, usually achieved via extreme caloric restriction. AN is complicated by malnutrition that can lead to life-threatening medical consequences as a result of multiple organ failure and immune system dysfunction^2,3^.

Starvation, malnutrition, altered dietary patterns, and single-nutrient deficiencies can all cause impaired immune functioning that can lead to chronic inflammation and recurrent infections^4,5^. Indeed, malnourished children chiefly die from “common infections”^4,6^. Obese individuals, who often have micronutrient deficiencies, experience more frequent and more severe infections^4^. Paradoxically, clinical observations of patients with AN report an absence of infections as well as delayed or reduced physiological responses to infection^7–11^. Moreover, AN associated dermatological changes include xerosis (dry skin), increased acne, slower wound healing, generalized pruritis, and seborrheic dermatitis, but an increased risk of skin infections has not been reported^12^. This is in striking contrast to an increased risk of skin infections associated with primary malnutrition typically seen in developing nations^13,14^. Cell-mediated immunity appears to be amplified in AN; however, this proinflammatory state does not sufficiently explain the lower incidence of infections^2,11,15,16^.

Antimicrobial peptides (AMPs) are evolutionarily conserved effector molecules of the innate immune system with broad-spectrum antimicrobial activities^17^. Psoriasin and RNase 7 are the most abundant AMPs found on human skin that serve immunomodulatory roles in skin immunity through the induction of cytokines and chemokines^18,19^. In the chronic skin inflammatory disease psoriasis, keratinocytes proliferate in response to inflammatory cytokines, which in turn increases the synthesis of AMPs, including psoriasin, and contributes to the recruitment of T cell subsets and other immune effector cells into the skin^20,21^. RNase 7 is induced by proinflammatory cytokines and a wide spectrum of potential pathogenic microorganisms such as *Staphylococcus aureus* and *Candida albicans*^19,22^. Similar to psoriasin, RNase 7 is upregulated in psoriasis and atopic dermatitis^23,24^. It is thus intriguing that the expression of AMP genes are also induced by starvation in *Drosophila melanogaster* (common fruit fly) in the absence of infection and independent of the pathogen-response pathway^25–27^. It is possible that this mechanism evolved to ensure innate immune activity during periods of energy deprivation.

Previously, to evaluate whether weight status may also affect AMP expression in human skin, we analyzed the concentrations of the AMPs psoriasin and RNase 7 on the skin surface of patients with AN before and after weight gain. Surprisingly, we found AMP concentrations did not decrease with weight gain, but rather an association of weight gain with increasing AMP concentrations was observed^28^. While a link between AN and skin immune function has yet to be elucidated, we hypothesize here that changes in the skin microbial profile of patients with AN might contribute to the absence of skin infections observed in this population.

In this study, we conducted an analysis of skin microbiota based on 16S rRNA gene amplicon sequencing in female patients with AN before and after undergoing an inpatient treatment program to gain weight and compared to age-matched healthy-weight controls. To test for possible relationships between AMP concentrations, bacterial load, or body mass index (BMI) and skin microbiota, and to gain insight into whether such relationships might contribute to the resistance to dermatological infections observed in AN, we analyzed skin microbial profiles in conjunction with these measures. We observe increasing levels of bacterial load with weight gain in patients with AN, which is significant at the inner elbow sampling location. In a collective analysis of the sampled body sites, we reveal a significant correlation between psoriasin concentrations and the healthy-weight control group indicator taxon *Abiotrophia*. In a similar analysis, we further find Shannon diversity significantly negatively correlates with psoriasin concentrations as well as total bacterial load. Psoriasin concentrations also significantly correlate with bacterial loads at the forehead sampling site across study groups. Finally, we observe a significant correlation between an individual’s BMI and *Lactobacillus*, a significant microbial indicator of health.

## Results

### Study participants and skin sampling

Thirty-three females diagnosed with AN receiving inpatient medical care and thirty-three healthy-weight age-matched female control subjects from Germany were recruited for this study (see “Methods” for inclusion and exclusion details). One patient with AN withdrew from the study prior to the second sampling point. Patient metadata analyzed in this study are summarized in Table 1; complete study metadata are provided online in Supplementary Table S1. In patients with AN, the mean BMI was 12.56 kg/m^2^ (SD 1.70) before weight gain and 14.54 kg/m^2^ (SD 1.70) after weight gain, with a mean weight gain of 5.7 kg (SD 1.50) corresponding to an increase in BMI of 2.0 (SD 0.50) points. The mean BMI of the healthy-weight control group was 22.10 kg/m^2^ (SD 1.73). All patients with AN had been diagnosed with a severe and life-threatening stage of AN, according to DSM-5 criteria, as represented by a BMI of 15.0 kg/m^2^ or less. Body mass index standard deviation scores (BMI SDS) in regard to age (years) is provided for the anorexia study cohort in Supplementary Table S2.

**Table 1:** Summarized metadata.

	AN (n = 33)	HC subjects (n = 33)
**Age at time of first measurement**		
Mean (SD)	25.80 years (9.90)	26.00 years (9.90)
Median	23.00	22.50
Range	17–54	16–57
**Weight (kg) first measurement, before weight gain**		
Mean (SD)	35.40 (5.2)	61.55 (6.18)
Median	36.90	61.00
Range	23.40–45.50	49–80
**BMI first measurement, before weight gain**		
Mean (SD)	12.56 (1.70)	22.10 (1.73)
Median	12.87	22.28
Range	9.22–14.8	18.69–25.84
	AN (n = 32)	HC subjects (n = 33)
**Weight (kg) second measurement, after weight gain**		
Mean (SD)	40.30 (5.20)	–
Median	42.40	–
Range	29–47	–
**BMI second measurement, after weight gain**		
Mean (SD)	14.54 (1.72)	–
Median	14.81	–
Range	10.73–17.55	–

*AN* patients with anorexia nervosa, *HC* healthy weight controls.

**Figure 1:**
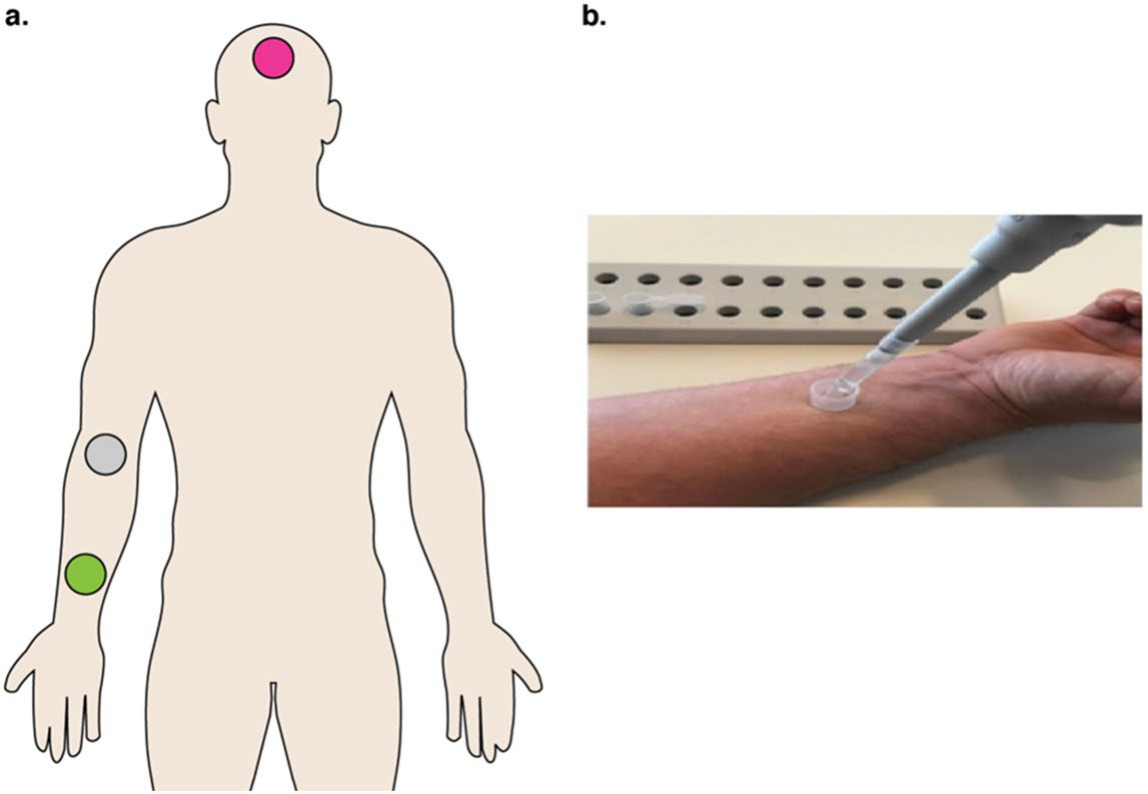
Overview of sampling procedure used in this study. (**a**) Standardized sampling locations for healthy-weight controls and patients with AN included the forehead, a sebaceous zone (pink), the antecubital fossa/ inner elbow, a moist zone (grey), and the ventral side of the lower arm, a dry zone (green). Patients were positioned accordingly to facilitate sampling procedures, e.g., supine for forehead sampling. Illustration by B. Hermes. (**b**) Sampling procedure of the distal (lower) forearm. Image photographed by Bendix et al.^28^.

To obtain skin bacterial profiles and bacterial load estimates, we extracted DNA from material derived from a skin-rinsing protocol that concurrently collected the antimicrobial peptides psoriasin and RNase 7 from three standardized body sites representing sebaceous, moist, and dry cutaneous zones (Fig. 1). Sampling sites were 1.77 cm^2^ in size and included the forehead (sebaceous), the antecubital fossa (referred to as inner elbow in this study; moist), and the ventral side of the lower forearm (dry). Patients were positioned accordingly to facilitate sampling procedures, e.g., were placed supine for forehead sampling.

For the subsequent analyses, we defined three study subject groupings: (1) healthy-weight controls (HC) defined by a BMI ranging between 18.5 and 25.0 kg/m^2^, (2) patients with AN prior to gain weight (hereafter referred to as AN before weight gain), and (3) patients with AN after undergoing an inpatient protocol to gain weight and after having gained at least 2 kg of body cell mass (hereafter referred to as AN after weight gain). The second sampling point for patients with AN was defined such that sufficient weight gain could be readily achieved by patients while under-going inpatient care (see “Methods” section). We expected an effect on biological parameters after a weight gain of one BMI unit. We analyzed these three groups according to total bacterial load derived from digital droplet PCR (ddPCR), the relative abundance of major taxa and diversity patterns identified in the 16S rRNA gene analysis, and our previously published concentrations of psoriasin and RNase 7^28^. A summary of the mean and median concentrations of psoriasin and RNase 7 is provided in Supplementary Table S3.

### Bacterial load

**Figure 2:**
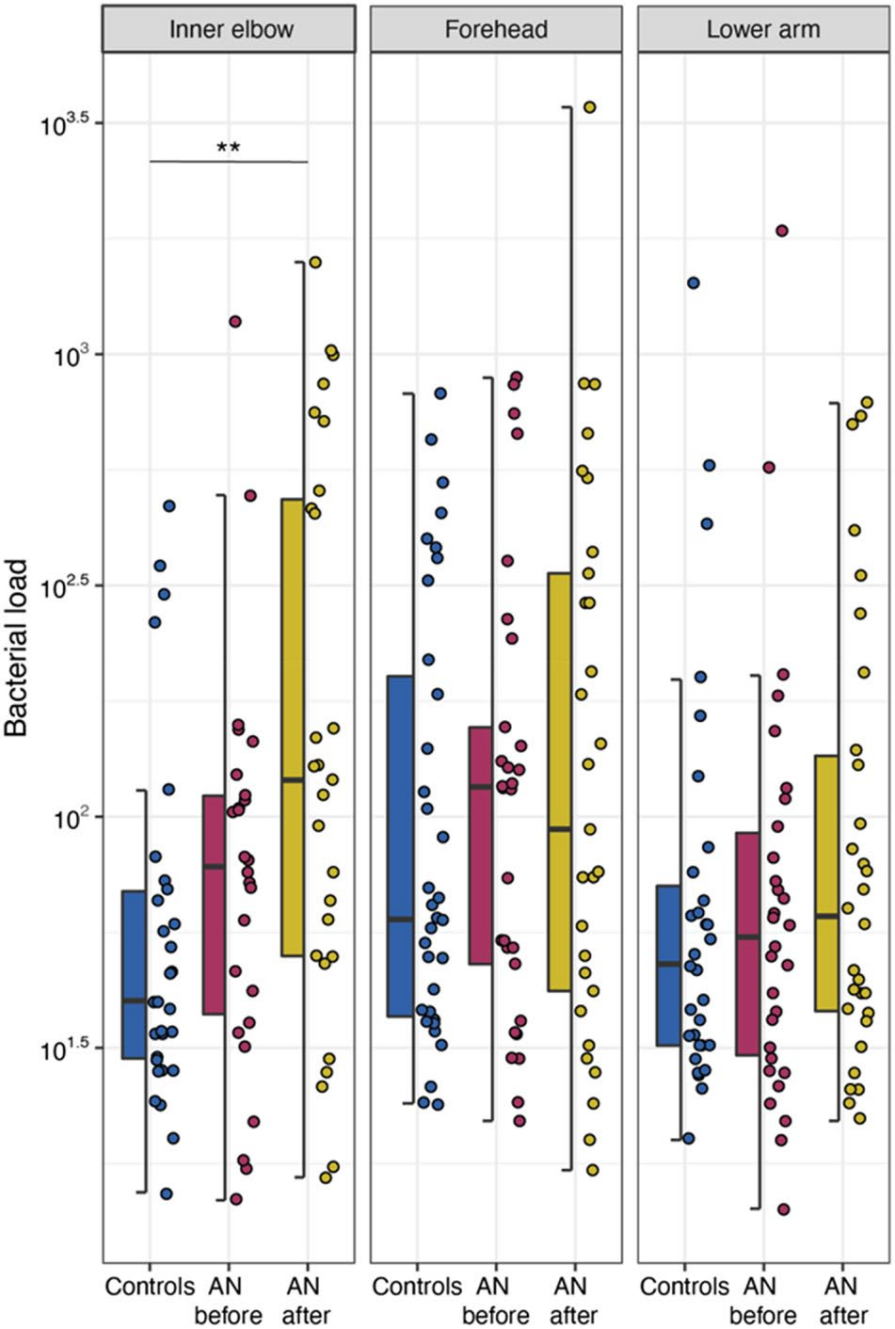
Box plots of total bacterial load, as measured by ddPCR, for study groups at individual sampling locations. AN = anorexia nervosa. Wilcoxon test (see “Methods” section); *p*-values: *< 0.05; **< 0.01; ***< 0.001. *p*-values were adjusted for multiple testing according to Benjamini and Hochberg^94^. Line indicates the median concentration; box shows the interquartile range (IQR), and the whiskers are 1.5 × IQR. Blue represents healthy-weight controls, red represents patients with AN before weight gain, and gold represents patients with AN after weight gain.

Due to the low microbial biomass of the skin environment and the associated technical challenges^29^, and the reasonable expectation that AMPs and/or AN disease status could influence bacterial load, we initially measured the total bacterial load of each sample using ddPCR to obtain a precise quantification of target DNA copies, as described by Sze and colleagues^30^ (see “Methods” section) (Supplementary Table S4). Digital droplet PCR is a method whereby a sample is fractionated into tens of thousands of individual droplets using a water–oil emulsion; PCR is then carried out within each droplet thereby providing reliable, absolute quantification of the target molecule, reducing PCR bias, and increasing signal-to-noise ratios, especially in low biomass samples such as skin^30–35^. We assessed the distribution of bacterial load between groups at individual sampling locations (Fig. 2). We observe an overall trend of increasing bacterial load with weight gain in patients with AN. However, we largely find that differences in bacterial load between groups are not significant, except for at the inner elbow (antecubital fossa). Here, differences in load between HC and patients with AN after weight gain reach statistical significance but differences are not significant between patients with AN before and after weight gain (Wilcoxon; *p* = 0.009; *p* = 0.053, respectively; Fig. 2). We additionally find that bacterial load significantly correlates with psoriasin concentrations at the forehead (Spearman; r_s_ = 0.281, *p* = 0.019), but not at the inner elbow or lower arm (Supplementary Table S4). Bacterial load did not significantly correlate with RNase 7 concentrations or BMI.

Next, we used ddPCR measurements to aid the assessment of potential contamination (see “Methods” section for a detailed description). Briefly, total bacterial load was used as a proxy for input bacterial DNA concentrations for the ‘frequency’ method within the R package *‘decontam’* (version 1.8.0; see “Methods” section)^36,37^. To verify amplicon sequence variants (ASVs) classified as contaminants (n = 154), we visualized five randomly selected contaminants in frequency plots to examine the distribution of the ASV with respect to bacterial loads. We find that the contaminant ASVs follow an expected pattern in which frequency is inversely proportional to bacterial load, as contaminating DNA will account for a larger fraction of this load in samples with low biomass^36^. We subsequently utilized the ‘prevalence’ method within *‘decontam’* (version 1.8.0), in which the prevalence (presence /absence) of ASVs in samples is compared to that in negative controls, to identify additional contaminants^36^. An additional 70 ASVs were identified as contaminants and removed from the dataset. Finally, following the recommendations of Weyrich et al.^38^, any ASV belonging to families *Halomonadaceae* (n = 0) or *Shewanellaceae* (n = 14) were removed. In total, we analyzed more than 400,000 sequences, with a normalized coverage of 1000 sequences per sample (see “Methods” section).

### Overview of skin microbiota in patients with AN and healthy-weight controls

**Figure 3:**
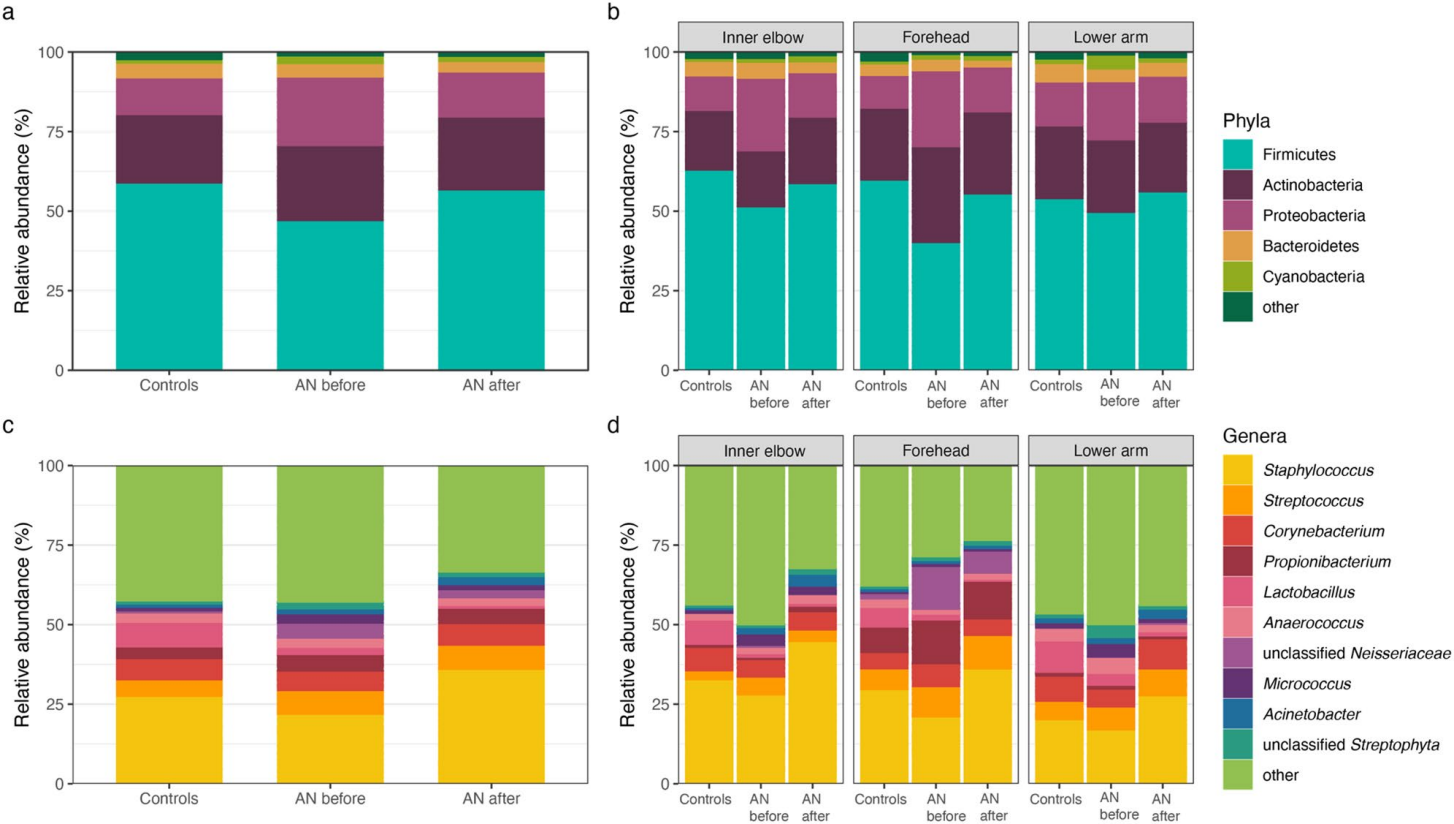
Overview of dominant taxa at sampling sites. (**a**) Bar plot of relative abundances for the most abundant phyla, and (**b**) at sampling sites (inner elbow, forehead, and lower arm). (**c**) Bar plot of relative abundances for the most abundant genera, and (**d**) at sampling sites (inner elbow, forehead, and lower arm). *AN* anorexia nervosa, *Controls* healthy-weight controls, *AN before* patients with AN before weight gain treatment, *AN after* patients with AN after weight gain treatment.

We first analyzed community composition at the phylum and genus levels. The dominant phyla include Firmicutes, Actinobacteria, Proteobacteria, Bacteroidetes, and the dominant genera include *Staphylococcus*, *Streptococcus, Propionibacterium*, *Corynebacterium*, *Anaerococcus,* and *Lactobacillus*, whose relative proportions are shown in Fig. 3. Comparisons of relative abundances between groups at the phylum level revealed significant differences in Proteobacteria between patients with AN before and after weight gain and between HC and patients with AN before weight gain (Wilcoxon; *p* = 0.005, *p* = 0.014, respectively). Additionally, we find significant differences in Firmicutes abundance between HC and patients with AN before weight gain (Wilcoxon; *p* = 0.003). At the genus-level, there are significant differences in the relative abundance of *Lactobacillus* between HC and AN before weight gain, as well as between HC and AN after weight gain groups (Wilcoxon; *p* = 4.23e−07, 5.12e−08, respectively). Other significant differences between groups include *Staphylococcus* for AN before compared to HC (Wilcoxon; *p* = 0.021) and AN before compared to AN after (Wilcoxon; *p* = 0.005), unclassified *Neisseriaceae* for both AN before and AN after weight gain compared to HC (Wilcoxon; *p* = 0.003, *p* = 0.026, respectively) and unclassified *Streptophyta* for AN after weight gain compared to HC (Wilcoxon; *p* = 0.049). Supplementary Table S5 provides a summary of statistical analyses comparing the most abundant phyla and genera between groups.

### Diversity indices

**Figure 4:**
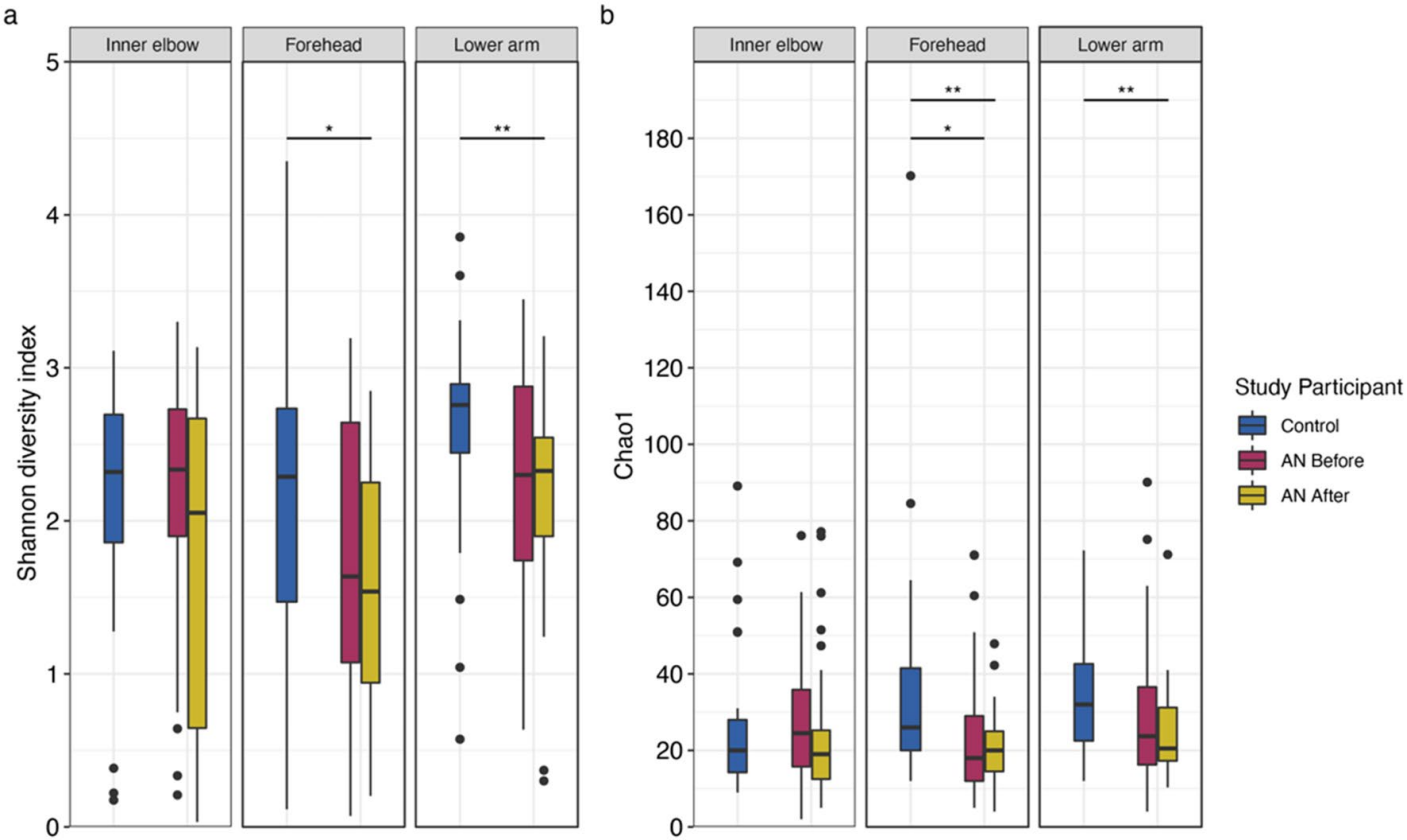
Alpha diversity indices for healthy-weight controls and patients with anorexia nervosa (AN), by weight gain status (before and after), at each sampling location. (**a**) Shannon diversity index. (**b**) Chao1 diversity index. Wilcoxon test (see “Methods” section); *p*-values: *< 0.05; **< 0.01; ***< 0.001. *p*-values were adjusted for multiple testing according to Benjamini and Hochberg^94^. Line indicates the median concentration; box shows the interquartile range (IQR), and the whiskers are 1.5 × IQR. Blue represents healthy-weight controls, red represents patients with AN before weight gain, and gold represents patients with AN after weight gain.

Next, we assessed alpha diversity at the ASV-level to investigate potential effects of AN on skin microbiota. Shannon diversity measures both the richness (number of different species) and evenness (how the species are distributed relative to one another) of the bacterial community, whereas the Chao1 index reflects expected species richness. Surprisingly, we show alpha diversity tends to decrease in patients with AN after weight gain therapy and find significant differences in both community richness and evenness in these patients compared to HC. Specifically, we find a significant difference in Shannon diversity at the forehead between HC and AN after weight gain (Wilcoxon; *p* = 0.018; Fig. 4a). We also find a significant difference in Shannon diversity at the lower forearm between HC and AN after weight gain (Wilcoxon; *p* = 0.005; Fig. 4a). For Chao1 diversity, we find a significant difference at the forehead between AN before weight gain and HC and between AN after weight gain and HC (Wilcoxon; *p* = 0.019; *p* = 0.007, respectively; Fig. 4b). As with Shannon diversity, there is a significant difference in Chao1 diversity at the lower forearm between HC and AN after weight gain (Wilcoxon; *p* = 0.009; Fig. 4b). Supplementary Table S6 provides summary statistics for group comparisons.

**Figure 5:**
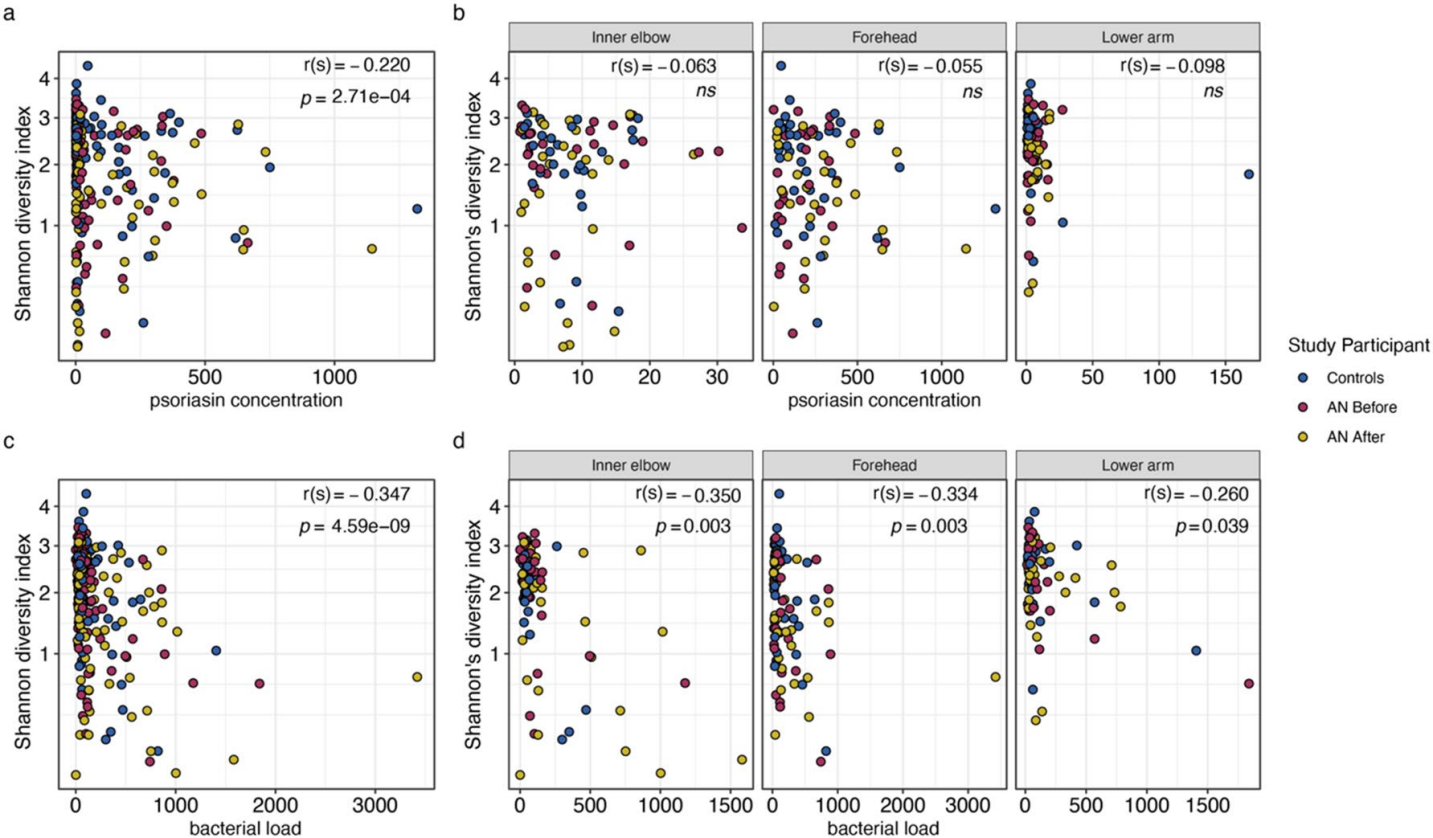
Spearman correlations between Shannon diversity index and (**a**) psoriasin concentrations, (**b**) psoriasin concentrations at individual sampling locations, (**c**) total bacterial load, and (**d**) total bacterial load at individual sampling locations. r(s) = spearman’s Rho. AN = anorexia nervosa. ns = not significant. Blue represents healthy-weight controls, red represents patients with AN before weight gain, and gold represents patients with AN after weight gain. *p*-values were adjusted for multiple testing according to Benjamini and Hochberg^94^.

To further explore the trend of decreasing alpha diversity after weight gain in patients with AN, we calculated Spearman correlations for Shannon and Chao1 diversity measures with AMP concentrations, total bacterial load, and BMI (Supplementary Table S7). We find that Shannon diversity is significantly negatively correlated with psoriasin concentrations (Spearman; rho = − 0.220, *p* = 2.71e−04; Fig. 5a), but not within individual sampling sites (Fig. 5b). We find Shannon diversity also significantly negatively correlates with total bacterial load (Spearman; rho = − 0.347, *p* = 4.59e−09; Fig. 5c), and moreover, this significant relationship is preserved at the inner elbow (Spearman; rho = − 0.350, *p* = 0.003), the forehead (Spearman; rho = − 0.334, *p* = 0.003), and the lower arm (Spearman; rho = − 0.260, *p* = 0.039; Fig. 5d). Shannon diversity does not significantly covary with RNase 7 concentrations or BMI. Chao 1 index does not significantly correlate with AMP concentrations, total bacterial load, or BMI.

To assess overall community compositional differences between groups, we next performed beta diversity analyses. We find no distinguishable separation of study groups based on the Bray–Curtis dissimilarity index (based on abundance) or with the Jaccard index (based on presence/absence), suggesting similar microbial communities amongst the groups (Supplementary Fig. S1). A constrained analysis of principal coordinates of the Bray–Curtis distance (‘*capscale’*^39^) with respect to treatment status (i.e., HC, AN before, and AN after) reveals significant differences between the study groups, but treatment status explains only about 1% of the variation between groups (anova.cca; *p* = 1.00e−04; Supplementary Fig. S2).

### Indicator species

To reveal potentially important individual taxa, we conducted indicator species analyses (‘*indicspecies’*^40^) at the ASV- and genus-level on a microbiota core defined by a prevalence threshold, whereby a taxon must be present in at least 5% of samples for inclusion in the analysis (see “Methods” section).

**Figure 6:**
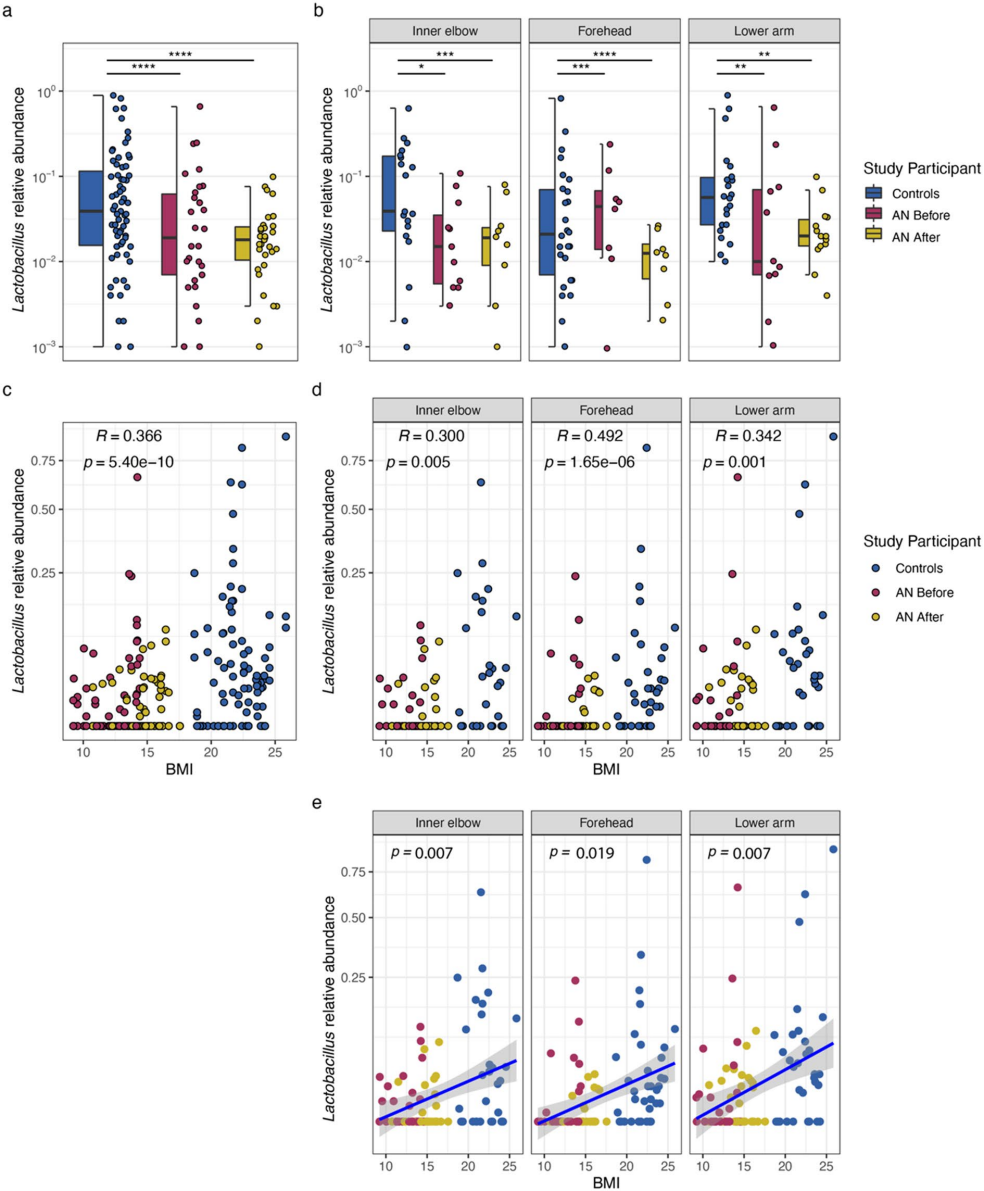
Box plots of relative abundances, Spearman correlations, and single linear regressions for *Lactobacillus*, an indicator genus. (**a**) Box plot of *Lactobacillus* relative abundances for healthy-weight controls and patients with AN by weight gain group (before and after) and, (**b**) faceted by sampling location. Wilcoxon test (see “Methods” section); *p*-values: *< 0.05; **< 0.01; ***< 0.001. Line indicates the median concentration; box shows the interquartile range (IQR), and the whiskers are 1.5 × IQR. (**c**) Spearman correlation between indicator *Lactobacillus* and BMI, and at (**d**) individual sampling locations (**e**) Single linear regression model between BMI and relative abundance of *Lactobacillus* at the inner elbow (R^2^_adj_ = 0.085; *p* = 0.007)*,* the forehead (R^2^_adj_ = 0.048; *p* = 0.019), and at the lower arm (R^2^_adj_ = 0.076; *p* = 0.007). r(s) = spearman’s Rho. *AN* anorexia nervosa, *BMI* body mass index. Blue represents healthy-weight controls, red represents patients with AN before weight gain, and gold represents patients with AN after weight gain. *p*-values were adjusted for multiple testing according to Benjamini and Hochberg^94^).

At the genus-level, *Lactobacillus*, *Clostridium sensu stricto*, and *Abiotrophia* associate with the HC group (Table 2). Accordingly, there is a statistically significant difference in the relative abundance of *Lactobacillus* in HC compared to both AN before and AN after weight gain groups (Wilcoxon; *p* = 4.23e−07, *p* = 5.12e−098, respectively; Fig. 6a; Supplementary Table S5). These significant differences are maintained at individual sampling locations (Fig. 6b; Supplementary Table S5). Further, we identify unclassified *Neisseriaceae* as a significant indicator for both the AN before and AN after groups. Summary statistics for differences in indicator genera between groups, and between groups at individual sampling locations, are presented in Supplementary Table S5.

**Table 2:** Indicators at genus- and ASV-level with RDP SeqMatch results.

	Stat	*p*-value	adj. *p*-value	RDP SeqMatch result	S_ab score
**Healthy controls**					
*Lactobacillus*	0.706	2.00E−05	0.002	*Lactobacillus crispatus*	1.00
ASV_29	0.706	4.00E−05	0.005		
*Abiotrophia*	0.404	9.00E−04	0.030	*Abiotrophia defectiva*	1.00
ASV_160	0.404	0.001	0.028		
*Clostridium *sensu stricto	0.374	0.001	0.030	*Clostridium* spp.	1.00
ASV_744	0.374	0.001	0.028		
**Patients with AN**					
unclassified Neisseriaceae	0.502	0.002	0.047	NA	NA
ASV_13	0.502	0.003	0.049		

Indicator species analysis applied using *‘indicspecies’* (version 1.7.9) with “r.g.” function and 99,999 permutations on a microbial core defined by ASVs classified to the genus-level that are present in at least 5% of all samples (see “Methods” section). *p*-values were adjusted for multiple testing according to Benjamini and Hochberg^94^.

Representative 16S rRNA gene sequences were queried via Ribosomal Database Project SeqMatch (version 3).

S_ab scores represent the number of unique 7-base oligomers shared between the query sequence and a given RDP sequence for both type- and non-type strains. The higher the S-ab score, the better the match.

*ASV* amplicon sequent variant, *RDP* ribosomal database project, *AN* anorexia nervosa.

Subsequently, because the *‘decontam’* procedure was performed on the level of ASVs, we conducted an additional screen to evaluate whether indicator genera could represent contaminants in our dataset, based on the expectation of negative correlations between bacterial load and contaminating taxa^36^. We thus accordingly calculated Spearman correlations between the relative abundance of indicator genera and bacterial loads. We find no significant correlations between total bacterial load and *Lactobacillus*, *Abiotrophia*, *Clostridium sensu stricto*, or unclassified *Neisseriaceae* (see “Methods” section; Supplementary Table S8). However, our analysis finds *Jeotgalicoccus*, an additional indicator genus for the HC group, to negatively correlate with total bacterial load (Spearman; r_s_ = − 0.129, *p* = 0.034). This association is not significant at individual sampling locations. Nevertheless, following the logic that contaminant sequences are expected to negatively covary with bacterial loads, *Jeotgalicoccus* was excluded from additional analyses and not reported in Table 2.

At the ASV level, we find three significant indicators for the HC group, and one for both the patients with AN before and after weight gain (Table 2). To refine the taxonomic classification of indicator ASVs, we queried representative sequences using RDP SeqMatch (see “Methods” section; Supplementary Table S9). Indicator ASV_29 is a close match to *Lactobacillus crispatus* (S_ab score = 1.00)*. Lactobacilli* spp. are well-known human commensals, with previous studies reporting *Lactobacilli* spp. in the gut, vagina, mouth, on skin, and in breastmilk^41–43^. A query of indicator ASV_160 reveals a close match to *Abiotrophia defectiva* (S_ab score = 1.00). Previous studies identified *Abiotrophia* spp., from the family *Lactobacillales*, in the oral and upper respiratory flora^44^. *Clostridium sensu stricto* was identified in the human gut microbiome in the context of chronic disease^45^ and was previously classified as a human-associated microbe with pathogenic capabilities^46^.

To evaluate potential associations between the relative abundance of indicator genera with AMP concentrations and BMI, we calculated Spearman correlations (see “Methods” section; Supplementary Table S8). We find that *Abiotrophia* significantly positively associates with psoriasin concentrations (Spearman; r_s_ = 0.174, *p* = 0.004; Supplementary Fig. S3). However, at individual sampling locations, i.e., inner elbow, forehead, and lower arm, these correlations are not significant (Supplementary Fig. S3). *Abiotrophia* does not significantly correlate with RNase 7 concentrations or BMI. We find that *Lactobacillus* does not correlate with psoriasin or RNase 7 concentrations. However, we find significant associations between BMI and *Lactobacillus* (Spearman; r_s_ = 0.366, *p* = 5.42e−10; Fig. 6c); further, this significant association is maintained at the inner elbow (Spearman; r_s_ = 0.300, *p* = 0.005), the forehead (Spearman; r_s_ = 0.492, *p* = 1.65e−06), and lower arm (Spearman; r_s_ = 0.342, *p* = 0.001; Fig. 6d). Since *Lactobacillus* is an abundant taxon, we selected it to conduct single linear regression modeling to assess whether BMI predicts the relative abundance of *Lactobacillus*. We find BMI to be a weak, but significant predictor of *Lactobacillus* relative abundance at the inner elbow (R^2^_adj_ = 0.085; *p* = 0.007)*,* the forehead (R^2^_adj_ = 0.048; *p* = 0.019), and at the lower arm (R^2^_adj_ = 0.076; *p* = 0.007; Fig. 6e).

## Discussion

Our study is the first to characterize the skin microbiota in female patients with AN. We conducted a 16S rRNA gene-based analysis in patients with AN before and after weight gain and with age-matched, healthy-weight controls and then correlated these findings with the concentrations of two highly abundant skin AMPs, psoriasin and RNase 7, and with BMI.

Notably, we find that the concentration of the AMP psoriasin weakly, but significantly, correlates with the indicator genus *Abiotrophia* for HC. However, at individual sampling locations, this genus does not significantly correlate with psoriasin concentrations, possibly due to low frequencies and low relative abundances.

Recently, *Abiotrophia* was found to be positively associated with the severity of psoriasis, a mixed autoimmune and autoinflammatory skin disorder marked by elevated psoriasin concentrations^47^. In this regard, our findings that *Abiotrophia* significantly positively associates with psoriasin, an established biomarker for psoriasis, supports evidence that links *Abiotrophia* and psoriasis disease severity. In our study, the relative abundance of *Abiotrophia* represents less than 1% of the total abundance of skin microbiota. This is fitting, as our study population did not exhibit signs of inflammatory skin disease or psoriasis, and thus, we would not expect this taxon to be a dominant genus. Still, it is interesting that *Abiotrophia* is an indicator for HC, but not for patients with AN, where one might expect inflammation to occur alongside AN-associated skin changes. The role of cell-mediated immunity in AN is controversial, with several studies reporting an increase in T-cell proliferation and inflammatory cytokine production, including interleukin-1, interleukin-6, and tumor-necrosis factor, when compared to healthy controls or to innate immunity responses in primary malnutrition, where immune function is suppressed ^2,11,15,16^. However, an earlier investigation conducted by Omodei and colleagues^48^ found that immune cell populations and the cytokines they produce are reduced in AN, but display greater antioxidant, stress resistance, and anti-inflammatory profiles compared to controls. It is possible that the AN population included in our study exhibits an augmented anti-inflammatory profile, thereby clarifying the relatively reduced levels of microbial taxa associated with inflammatory skin disease, such as *Abiotrophia*, observed in these subjects.

We also find the Shannon diversity index, which reflects both species richness and species evenness, to significantly negatively associate with psoriasin concentrations and with total bacterial load. Furthermore, we show that both Shannon and Chao1 diversity decreases in patients with AN after weight gain compared to baseline weight and compared to HC, and that there are significant differences in alpha diversity between HC and AN after weight gain.

These results are interesting in the context of our previous work, involving the same study cohort, in which we found that AMP concentrations, and psoriasin in particular, tended to increase in patients with AN after weight gain^28^. The relationship between the antimicrobial peptide psoriasin and skin microbiota is still unclear. Previous surveys of patients with psoriasis report higher alpha diversity, but with lower stability, compared to healthy skin^49^. However, other studies report decreased taxonomic diversity in psoriatic skin compared to healthy skin^50^. These data are notable as increased psoriasin expression is a well-established feature of psoriasis^51^. We propose that the observation of increasing psoriasin concentrations with weight gain in severely underweight patients may in turn reduce taxonomic diversity on the skin, perhaps through targeted bactericidal activity^28,52^. It is possible that our findings capture a shift in alpha diversity in low weight patients in response to rising psoriasin levels.

Additionally, we find that BMI significantly correlates with *Lactobacillus*, another indicator genus for HC. Since *Lactobacillus* represents a dominant genus in our study, we assessed the possibility that *Lactobacillus* is a contaminant. We visualized the distribution of *Lactobacillus* across bacterial loads obtained from ddPCR for the most abundant *Lactobacillus* ASVs in our dataset. We find that these *Lactobacillus* ASVs do not follow a pattern of contamination, whereby their frequency would be inversely proportional to input bacterial load. Rather, the frequency of *Lactobacillus* ASVs are independent of the input ddPCR load data. These findings are consistent with *Lactobacillus* representing a true biological signal.

The finding of *Lactobacillus,* and *L. crispatus* in particular, as an indicator of healthy-weight is congruent with previous studies demonstrating the potential role of *Lactobacilli* spp. as probiotics for improving skin health and barrier function^53,54^, anti-aging effects^55^, and balancing the gut microbial population, thereby preventing inflammatory disease and even cancer at different sites in the body, most likely through the production anti-inflammatory metabolites such as short chain fatty acids^56^. Moreover, this finding is supported by data showing that *Lactobacillus* colonizes healthy human skin^57–61^, including the inner elbow^62^, forehead^63^, and scalp^64^. Interestingly, one study exploring the effects of age on the structure of the skin microbiome found *Lactobacillus* to be present on the skin of participants aged 20–30 years, but not on those aged 50–60 years^65^. This finding is particularly interesting given that the mean age of our study population is 25 years for patients with AN and 26 years for HC. However, another survey of the skin microbiome in relation to age and photodamage found increasing age is associated with an increase in *Lactobacillus*^61^. Li et al.^61^ hypothesize that *Lactobacillus* spp. may increase in response to skin damage (e.g., from UV radiation) that accumulates with age, which may reduce inflammation and improve skin barrier integrity. Moreover, the authors speculate that *Lactobacillus*, *Staphylococcus*, and *Propionibacterium* (*Cutibacterium*) might act synergistically in skin immunity functions to protect and repair skin from photodamage and inflammation^61^.

In our study, it is possible that the skin microbiome is responding to inflammation and AN-associated skin changes in patients with AN as nutritional status improves. Although not significant, we find *Lactobacillus* to increase in patients with AN with weight gain at the inner elbow and lower arm. Conversely, *Lactobacillus* decreases at the forehead, but here, we also find increasing total bacterial load, which significantly correlates with increasing psoriasin concentrations, and we further observe a non-significant increase in *Staphylococcus* at the forehead (Supplementary Fig. S3, Supplementary Table S5). The synergistic actions of *Lactobacillus*, *Propionibacterium*, and *Staphylococcus* to mitigate inflammation and repair skin integrity could perhaps explain why we did not observe significant differences between patients with AN before and after weight gain when analyzing these taxa individually. Indeed, when we visualized the sums of the relative abundances for *Lactobacillus*, *Propionibacterium*, and *Staphylococcus*, we observe a significant difference in relative abundance between patients with AN before and after weight gain and between HC and patients before weight gain. Notably, the sum of these relative abundances in patients with AN after weight gain is not significantly different from HC (Supplementary Fig S4).

Lastly, our findings contribute to the growing body of evidence demonstrating that BMI significantly associates with skin microbial diversity. In a study of underweight (BMI 15–18.5), normal-weight (BMI 18.5–25.0), overweight (BMI 25–30), and obese individuals (BMI 30.0–45.0), Brandwein et al*.*^66^ found that skin microbial diversity was significantly associated with BMI. Specifically, the authors reported a significant difference in skin microbial diversity between underweight and overweight/ obese individuals and between underweight and normal-weight individuals, but not between normal-weight and overweight/ obese individuals. While limited in terms of sample size and weight categories (underweight and normal-weight only), our work supports the finding that features of the human skin microbiome covary with BMI.

Our study has some limitations. The range of diverse microenvironments (i.e., dry, moist, sebaceous) encompassing human skin as well as the need to consider bacteria living on the skin’s surface and those residing within its deeper layers introduce challenges^67–72^. Our sampling strategy included a skin washing method that, to our knowledge, has not been implemented in other skin microbiome surveys. This method is advantageous in that it allows for simultaneous collection of AMPs. A potential downside to this method is that the washing solution likely collects only superficial microbes that can be readily flushed off the skin using the rinsing solution. It is therefore possible that our findings are not necessarily comparable to other surveys of the skin microbiome in which methods such as skin scraping or swabbing, that can perhaps collect greater numbers of bacteria and bacteria at various depths, were utilized. This is perhaps evident in our bacterial load findings. Here, we believe the zero bacterial load scores for some samples represents values below the detection limit of our ddPCR method for particularly low biomass skin microbiota samples. Additionally, we find *Propionibacterium* at the oily forehead location in relative abundances less than that typically found in other skin microbiome surveys^73^. This finding may be a result of the rinsing solution not efficiently washing off *Propionibacterium*, which are known to adhere to free fatty acids on the skin^73^. Additionally, it is possible that other bacteria, such as *Lactobacillus*, are more readily washed off the skin’s surface and thus may be overrepresented in our results.

Our unique study population is also likely to influence the skin microbial profiles reported here. Our study included young women, including women under 18 years of age, with severe and life-threatening anorexia nervosa. Previous surveys found that pre-pubescent children often harbor low levels or no *Propionibacterium* on the skin^74,75^. A common side effect of malnutrition in severe and life-threatening anorexia nervosa is pubertal delay^76^. Thus, it is possible that the relative abundances of *Propionibacterium* in our study reflect those reported in surveys of pre-pubescent children. Moreover, a survey comparing skin microbiota profiles of hands between men and women found that taxa from *Lactobacillaceae* are more abundant on the hands of women^77^. The abundance of *Lactobacillus* reported in our study might reflect a larger phenomenon in which young women harbor greater abundances of these commensal bacteria on the skin compared to men. Future studies, especially those comparing the skin washing method with other established methods in the field, are necessary to verify these hypotheses.

Further, the timing of the second sampling point for AMP and skin microbiota collection may have occurred too soon to sufficiently capture additional meaningful changes in the composition of the skin microbiota. The skin microbiome is remarkably stable at the strain level, despite an ever-changing environment, and the composition of the skin microbiome is largely shaped by host physiology^78^. Given that the patients with AN were still significantly under-weight at the second sampling timepoint, with a mean BMI of 14.54 kg/m^2^, it is possible that any immune dysregulation affecting microbial composition at the first timepoint was still present after weight gain. Interestingly, Gibson et al.^2^ speculate that the proinflammatory state in AN is perhaps a primary immunity defect that contributes to the pathogenesis of AN. If immune dysregulation in AN is not necessarily secondary to malnutrition, then it stands to reason improvements in weight and nutrition status in patients with AN would not necessarily affect skin immune processes, and therefore may not lead to substantial changes in skin microbiota. Moreover, it is also feasible that the modest weight gain (at least 2 kg) in patients with AN between sampling timepoints one and two was not enough to alter skin physiology in other ways (e.g., increase sebum production), and therefore not enough to significantly alter microbial community structure. Finally, it is possible that the effect of starvation on AMP levels observed in *Drosophila* is not readily translated to humans^27^. Nutritional status and dietary intake affect human physiological and biochemical processes, yet little is known about the effect of nutrition on human skin physiology^79,80^.

In conclusion, this study explored whether changes in the skin microbial profile of patients with AN might contribute to the absence of skin infections observed in this population. Within the anorexia nervosa cohort, we do not observe an association between skin microbiota features and antimicrobial peptide concentrations. Moreover, we are unable to identify indicator species or other features of the skin microbiota in patients with AN that might provide an explanation for why this population seems to experience less frequent and less severe infections compared to other undernourished populations^4,5,7–11^. We do, however, find significant statistical correlations between the highly abundant skin AMP psoriasin and features of the skin microbiome of healthy-weight controls. Further investigation of the relationship between psoriasin and skin microbiota in the context of both healthy and diseased states is warranted.

Finally, we reveal a significant statistical correlation between an individual’s BMI and *Lactobacillus*, a significant microbial indicator of health, at all sampling locations, affirming a previously identified association between BMI and skin microbial diversity^65^. Further studies examining the relationship between caloric and nutritional intake and skin microbiota in the context of eating disorders may help clarify the physiological mechanisms that link nutritional intake with skin physiology.

## Methods

### Study subjects

The study was approved by the ethics committee of Hannover Medical School (3209-2016) and was conducted following the Declaration of Helsinki and in accordance with relevant guidelines and regulations. All participants or legal guardians provided written informed consent prior to study inclusion.

Thirty-three female patients diagnosed with AN according to DSM-5 criteria^81^, and with a body mass index (BMI) of 15 kg/m^2^ or below were recruited from two inpatient eating disorder facilities in Germany (Klinik Lüneburger Heide and Hannover Medical School). The DSM-5 defines AN by (a) a restriction of energy intake leading to a significant low body weight, (b) an intense fear of gaining weight or becoming fat, and (c) an unduly influence of body weight or shape on self-worth. Patients with AN were investigated shortly after hospitalization, prior to undergoing an inpatient treatment program to gain weight, and again after having achieved an increase in body cell mass of 2 kg or more. The second skin sampling time point was strategically defined. Full weight restoration in adult patients takes years to achieve and is not usually accomplished during inpatient care. Adult patients are typically discharged from inpatient care when they are still underweight, but after some degree of weight gain or symptom reduction has been achieved. Outpatient treatment usually follows discharge. Further, even modest weight gain, e.g., 2 kg, can be a therapeutic challenge in low weight patient populations. Moreover, we expected an effect on biological parameters after a modest weight gain of one BMI unit. Thus, an increase in body cell mass of 2 kg or more, which was deemed to be both therapeutically appropriate and achievable within a reasonable timeframe while still undergoing inpatient therapy, was defined as the second sampling point. One patient with AN withdrew from the study prior to the second sampling point. Randomly selected healthy-weight control subjects, defined by a BMI between 18.5 and 25.0 kg/m^2^, included thirty-three age-matched females without a psychiatric history and free of current mental disorders. Controls were investigated at one time point. Inclusion criteria for all subjects included a minimum age of 16 years, non-smoking status, and to be visually free from skin disorders. All subjects were free from inflammatory disease and immunosuppressive drugs. Final sample sizes included 287 skin rinsing samples from 32 patients with AN and 33 control subjects (Table 1; Supplementary Table S1).

Subjects underwent a clinical interview to gather socio-demographic information and medical history. Subjects were weighed using a standardized scale. BMI was calculated using height and weight data. Bioelectrical impedance analysis (BIACORPUS RX 4004 M, Medical Healthcare GmbH, Karlsruhe, Germany) was used to verify an increase in body cell mass of at least 2 kg prior to the second sampling point for the patients with AN.

### Sampling procedures

A standardized sampling procedure was implemented by Bendix et al*.*^28^, whereby sampling was conducted in the same location by one investigator at the same time to minimize putative influences of the circadian rhythm^82^. Three standardized body sites measuring 1.77 cm^2^ in size and comprising diverse skin microenvironments (i.e., sebaceous, moist, and dry body regions) were selected: forehead (sebaceous), inner elbow (moist), and ventral side of the lower forearm (dry; Fig. 1). All subjects avoided cosmetics, lotions, and other topical products the morning of testing. Subjects abstained from physical exercise the morning of sampling days, as exercise may increase AMP expression^83^. Subjects were placed in appropriate positions to collect samples from the various locations. For example, forehead sampling could be carried out with the subject placed in a supine position. Negative sampling controls including ambient air, room controls, and/or negative extraction controls were included for each sampling batch. Ambient air controls containing aliquots of the rinsing buffer solution used at the study site were opened and closed quickly, and then processed as samples. Room controls containing aliquots of the rinsing buffer solution used at the study site were left open for the duration of the rinsing procedure before being processed.

AMPs investigated in this study included psoriasin and RNase 7, which represent the two most abundant AMPs on the surface of human skin^84,85^. AMP data analyzed in this study was previously reported by Bendix et al*.*^28^. AMP sampling was conducted using a skin rinsing method, previously described by Bendix et al*.*^28^, Gläser et al*.*^85^, and Wittersheim et al*.*^86^. Briefly, standardized skin sites were washed by pipet with 1 ml of a rinsing buffer solution (10 mM sodium phosphate buffer, pH 7.4 containing 0.1% Triton X-100) using a sterile, DNA-free plastic ring. The buffer solution was collected, centrifuged (10 min, 10.000×*g*), and diluted 1:10 with 10% (w/v) bovine serum albumin. Samples were stored at −80 °C until further processing. Quantitative determination of the AMPs was measured by ELISA, as previously described by Gläser et al*.*^87^ and Bendix et al*.*^28^. A monoclonal antibody derived from hybridoma mouse cells was used for the psoriasin ELISA^85^. A polyclonal antibody derived from goat was used for the RNase 7 ELISA^84^. ELISA was performed twice for each sample to ensure reliability. A mean value was calculated from the two sampling measurements and subsequently used in downstream analyses.

Bacteria were collected concurrently with antimicrobial peptides during the skin rinsing protocol and harvested by centrifugation (10 min, 10.000×*g*). Bacterial genomic DNA was extracted from the resulting pellet formed during the centrifugation step of the skin rinsing procedure using the Ultra-Deep Microbiome Prep extraction kit according to the supplier’s protocol. Samples were extracted in batches corresponding to collection dates and library preparation and subsequent sequencing (see below) was completed in two batches (Supplementary Table S1).

### Bacterial load assessment and 16S rRNA gene sequencing

We adapted ddPCR to measure bacterial loads by targeting the V2 hypervariable region of the 16S rRNA gene, as described by Sze and colleagues^30^. The 20µL ddPCR master mix was prepared according to the manufacturer’s protocol with a final primer concentration of 120 nM and with 10 ng of nucleic acid template. PCR was performed on Bio-Rad C1000 Touch Thermal Cycler with following conditions: 95 °C for 5 min, 40 cycles at 95 °C for 15 s and 60 °C for 1 min, 4 °C for 5 min, 90 °C for 5 min, and incubation at 10 °C. Final products were transferred to QX200™ Droplet Reader and quantified as gene copies (per 20µL) using Bio-Rad QuantaSoft (v.1.7.4.0917).

16S rRNA amplicon library preparation and sequencing were performed as described in Belheouane et al*.*^88^ Briefly, hypervariable regions V1-V2 of the bacterial 16S rRNA gene were amplified and sequencing was performed using the dual-index sequencing strategy for amplicon sequencing on the Illumina MiSeq platform^89^. Negative controls were included in library preparation and sequencing batches. After PCR amplification, the final number of negative controls that were included for sequencing included: ambient air (n = 5), room controls (n = 5), and negative extraction controls with and without rinsing buffer (n = 16, respectively). All controls were processed alongside samples. ZymoBIOMICS Microbial Community Standard cells (Zymo Research) were used as extraction and sequencing controls to assess contamination^36^.

### Data processing and 16S taxonomic classification

Data processing and statistical analyses were performed in R (version 4.0.5). Processing and taxonomic classification of 16S rRNA gene sequence data was performed as previously described^88^. Sequences were processed using DADA2 (version 1.16.0), resulting in ASV abundance tables^90^. Taxonomic assignment of ASVs was completed in *‘DADA2’* with the Bayesian classifier using the NR Silva database training set, version 138^91^.

### Contamination assessment

As the skin harbors relatively low microbial biomass, the risk of contamination during skin sampling and sample processing is substantial and any contamination introduced during these steps can radically affect data interpretation, as contaminants tend to be preferentially amplified and sequenced over true biological signals within the sample ^29,36,92^. Here, we present a detailed description of steps taken throughout this study to mitigate and assess the contribution of contamination.

ddPCR load measurements were used to assess contamination in our dataset via the ‘frequency’ method within the R package *‘decontam’* (version 1.8.0) in conjunction with *‘phyloseq’* (version 1.32.0)^36,37,93^. The strict probability threshold of 0.1 was used and the batch feature within *‘decontam’* was utilized to analyze samples according to extraction batch to account for any batch effects and differences in contamination between batches. This resulted in the identification and subsequent removal of 154 ASVs labelled as likely contaminants. To verify ASVs classified as contaminants by the ‘frequency’ method within *‘decontam’*, we visualized five randomly selected contaminant ASVs in frequency plots to view the distribution of the ASV with respect to total bacterial loads obtained from ddPCR. We find that the ASVs identified as contaminants follow the expected pattern in which frequency is inversely proportional to input ddPCR load, as contaminating DNA will account for a larger fraction of the ddPCR load in samples with low biomass.

Next, we utilized the ‘prevalence’ method within *‘decontam’*, in which the prevalence (absence/ presence) of sequence features in samples is compared to the prevalence in negative controls to identify contaminants. The threshold parameter was set to the strict probability threshold of 0.5 and the batch function was utilized to analyze samples according to extraction batch and to account for differences in contamination between batches. An additional 70 ASVs were identified as likely contaminants and subsequently removed from the dataset. Finally, following recommendations of Weyrich et al*.*^38^, any ASV belonging to families *Halomonadaceae* (n = 0) and *Shewanellaceae* (n = 14) were removed, as these bacteria represent common contaminants in low biomass samples.

To normalize sequencing coverage after the *‘decontam’* filtering procedure, we calculated rarefaction curves to determine the sampling threshold; random sub-sampling to 1000 sequences per sample was performed. Twenty-three samples did not meet the 1000 sequences coverage threshold and were excluded from the analysis. Additionally, 761 ASVs were removed because they were no longer present in any sample after random sub-sampling.

### Ecological and statistical analyses

Statistical significance was defined at 0.05 and below for all analyses. Alpha diversity was measured using Shannon and Chao1 indices with ‘*vegan*’ (version 2.5-6.) As data were not normally distributed, all three groups were compared against each other using paired (AN before versus AN after) and unpaired (AN before versus HC, AN after versus HC) Wilcoxon rank sum tests. Spearman correlations were performed to assess the relationship between alpha diversity and BMI. Overall differences between groups (beta diversity) were assessed using the Bray–Curtis dissimilarity index in Phyloseq; the ‘*vegan*’ package was used to conduct a constrained analysis of principal coordinates (‘*capscale*’), a hypothesis-driven ordination that limits the separation of the communities based on the variable tested, for which the ‘anova.cca’ function was applied to assess significance.

Between group relative abundances at the phylum- and genus-level were calculated in ‘*phyloseq*’ and compared using Wilcoxon rank sum tests. Spearman correlations were performed to assess the relationship between the relative abundances of individual taxa and BMI or concentrations of psoriasin or RNase 7 at individual sampling locations (i.e., forehead, inner elbow, lower arm). Correction for multiple testing was performed according to Benjamini and Hochberg method^94^.

Indicator species analysis was applied using *‘indicspecies’* (version 1.7.9) with the ‘r.g.’ function^95^ and 99,999 permutations on a microbial core defined by ASVs classified to the genus-level that are present in at least 5% of all samples. Significant indicator ASVs were selected after correction of *p*-values for multiple testing using the Benjamini and Hochberg method^94^. We additionally calculated Spearman correlations between significant indicator taxa at the genus-level and bacterial loads. In cases of contamination, contaminant features are usually inversely proportional to input DNA concentration, as contaminants tend to be preferentially amplified and sequenced over true biological signals within the sample^29,36,92^. We find no significant negative correlations between bacterial loads and indicator genera reported (see Results), suggesting that these indicator taxa likely represent true biological signal.

Representative 16S rRNA gene sequences were queried via Ribosomal Database Project SeqMatch (version 3)^96,97^. Results represent classification based on the RDP match score (S_ab), which is the number of unique 7-base oligomers shared between the query sequence and a given RDP sequence for both type- and non-type strains.

## Supplementary Information

Supplementary Figures.

Supplementary Tables.

## Data Availability

The data for this study have been deposited in the European Nucleotide Archive (ENA) at EMBL-EBI under accession number PRJEB47175 (https://www.ebi.ac.uk/ena/browser/view/PRJEB47175).

## Abbreviations

AN: Anorexia nervosa
AMP: Antimicrobial peptide
BMI: Body mass index
ASV: Amplicon-sequence variants
