# Supplementary material for: Skin microbiota analysis in patients with anorexia nervosa and healthy-weight controls reveals microbial indicators of healthy weight and associations with the antimicrobial peptide psoriasin
Source: Sci Rep. 2022 Sep 15;12:15515. doi: 10.1038/s41598-022-19676-6 (PMC9477808; doi:10.1038/s41598-022-19676-6)
Supplement: Supplementary file 1 — Supplementary Figures. [file 41598_2022_19676_MOESM1_ESM.pdf]

Skin microbiota analysis in patients with anorexia nervosa and healthy-weight controls reveals microbial indicators of healthy weight and associations with the antimicrobial peptide psoriasin

Hermes, Britt M.[1,2,3*], Rademacher, Franziska[4], Chung, Cecilia[1], Tiegs, Gisa[5], Bendix, Marie-Christin[6], de Zwaan, Martina[6], Harder, Jürgen[4*+], Baines, John F.[1,2*+]

Author affiliations

[1] Max Planck Institute for Evolutionary Biology, Plön, Germany

[2] Section of Evolutionary Medicine, Institute for Experimental Medicine, Kiel University, Kiel, Germany

[3] Lübeck Institute of Experimental Dermatology, University of Lübeck, Lübeck, Germany

[4] Department of Dermatology, University Hospital Schleswig-Holstein, Kiel, Germany

[5] Institute of Experimental Immunology and Hepatology, University Medical Center Hamburg-Eppendorf, Hamburg, Germany.

[6] Department of Psychosomatic Medicine and Psychotherapy, Hannover Medical School, Germany

[+] These authors jointly supervised this work: Harder, Jürgen and Baines, John F.

[*] Corresponding authors: hermes@evolbio.mpg.de; baines@evolbio.mpg.de; jharder@dermatology.uni-kiel.de

Abbreviations: anorexia nervosa (AN), antimicrobial peptide (AMP), body mass index (BMI), amplicon-sequence variants (ASV)

Supplementary Figures

- **Supplementary Figure S1.** Beta diversity
- **Supplementary Figure S2.** Constrained analysis of principal coordinates of the Bray–Curtis dissimilarity index
- **Supplementary Figure S3.** Spearman correlations between indicator *Abiotrophia* and psoriasin concentrations
- **Supplementary Figure S4.** Box plots of relative abundances of *Staphylococcus*, *Propionibacterium*, and the sum relative abundance for *Staphylococcus*, *Propionibacterium*, and *Lactobacillus*

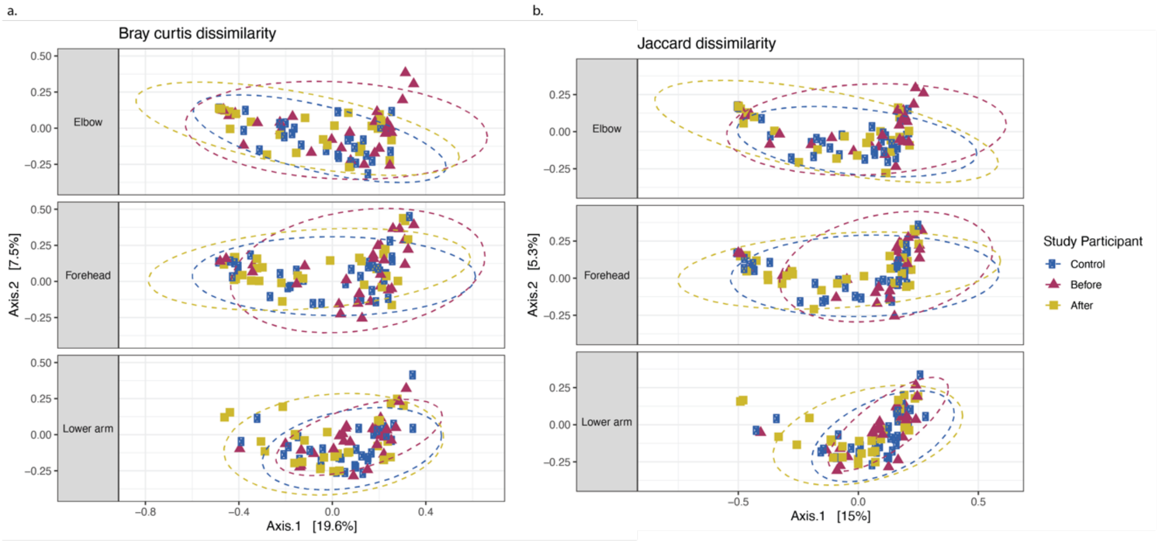

Supplementary Figure 1. Beta diversity based on a. Bray-Curtis dissimilarity index by sampling location and b. Jaccard index by sampling location. Blue represents healthy-weight controls, red represents patients with AN before weight gain, and gold represents patients with AN after weight gain. AN = anorexia nervosa

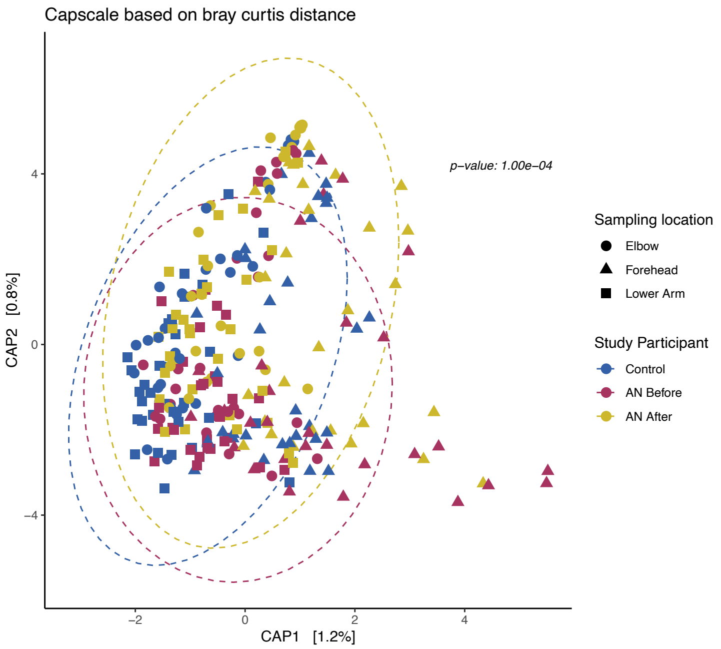

Supplementary Figure 2. Constrained analysis of principal coordinates of the Bray–Curtis dissimilarity index with respect to treatment status. Blue represents healthy-weight controls, red represents patients with AN before weight gain, and gold represents patients with AN after weight gain. AN = anorexia nervosa

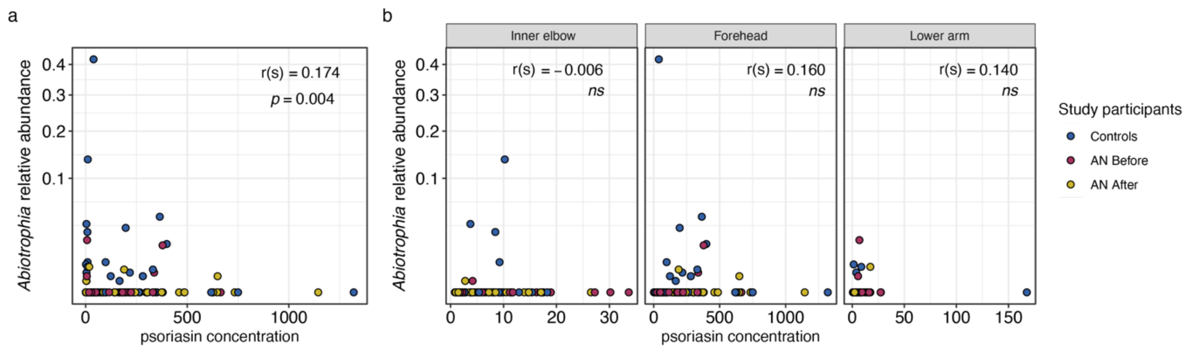

Supplementary Fig S3. Spearman correlations between indicator *Abiotrophia* and psoriasin concentrations a. at all locations and b. faceted by sampling location. r(s) = spearman's Rho. AN = anorexia nervosa. ns = not significant. Blue represents healthy-weight controls, red represents patients with AN before weight gain, and gold represents patients with AN after weight gain. *p*-values were adjusted for multiple testing according to Benjamini and Hochberg (1995).

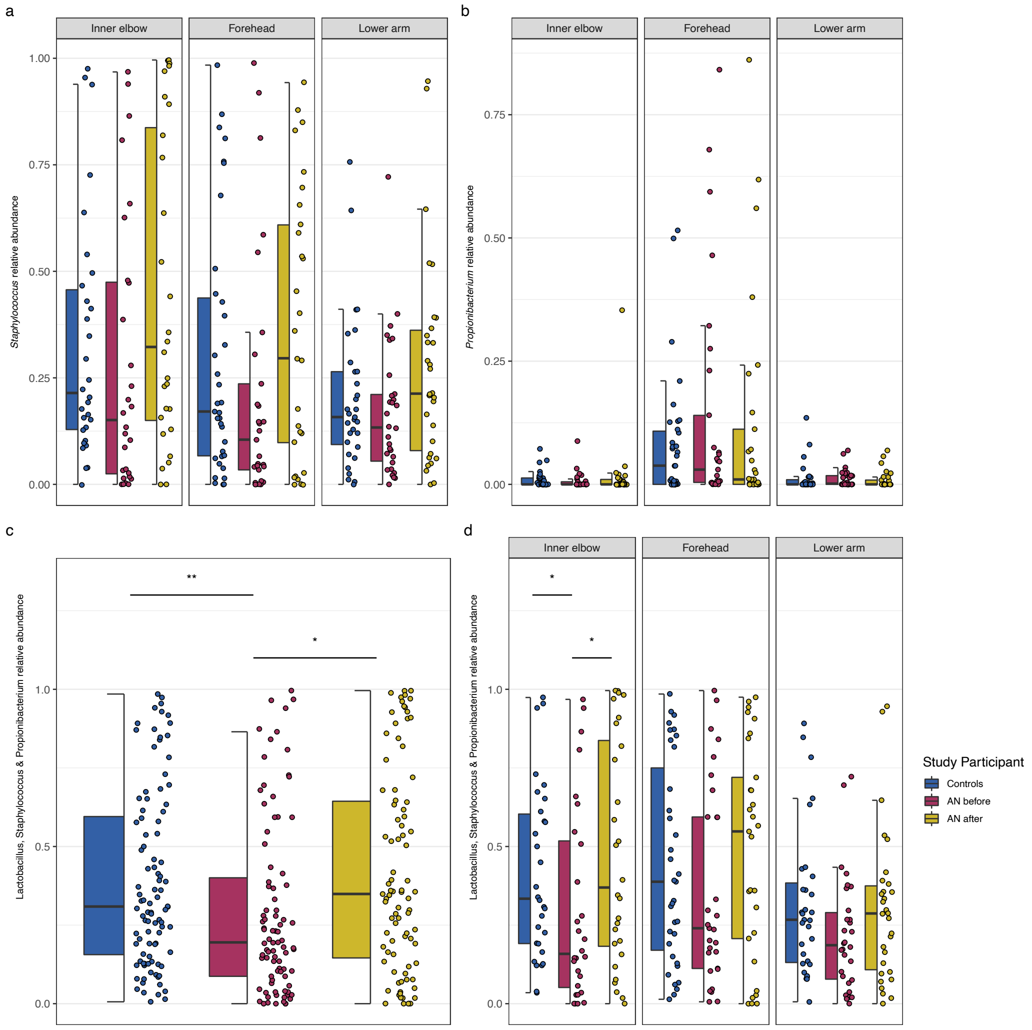

Supplementary Fig S4. Box plots of **a.** *Staphylococcus* and **b.** *Propionibacterium* relative abundances for healthy-weight controls and patients with AN, by weight gain arm (before and after) and, **c.** Sum totals of relative abundances of *Staphylococcus, Propionibacterium, and Lactobacillus* for healthy-weight controls and patients with AN, by weight gain arm

(before and after) and, **d.** faceted by sampling location. Wilcoxon test (see Methods**)**; *p*-values: * <0.05; ** < 0.01; *** < 0.001. *p*-values were adjusted for multiple testing according to Benjamini and Hochberg (1995). Line indicates the median concentration; box shows the interquartile range (IQR), and the whiskers are 1.5x IQR. Blue represents healthy-weight controls, red represents patients with AN before weight gain, and gold represents patients with AN after weight gain. Summary statistics provided in Supplementary Table S4. AN = anorexia nervosa
